# The illusion of the mind–body divide is attenuated in males

**DOI:** 10.1038/s41598-023-33079-1

**Published:** 2023-04-24

**Authors:** Iris Berent

**Affiliations:** Department of Psychology, Northeastern University, 125 Nightingale Hall, 360 Huntington Ave., Boston, MA 02115 USA

**Keywords:** Human behaviour, Social evolution, Psychology

## Abstract

A large literature suggests that people are intuitive Dualists—they tend to perceive the mind as ethereal, distinct from the body. Here, we ask whether Dualism emanates from within the human psyche, guided, in part, by theory of mind (ToM). Past research has shown that males are poorer mind-readers than females. If ToM begets Dualism, then males should exhibit weaker Dualism, and instead, lean towards Physicalism (i.e., they should view bodies and minds alike). Experiments 1–2 show that males indeed perceive the psyche as more *embodied*—as more likely to emerge in a replica of one’s body, and less likely to persist in its absence (after life). Experiment 3 further shows that males are less inclined towards Empiricism—a putative byproduct of Dualism. A final analysis confirms that males’ ToM scores are lower, and ToM scores further correlate with embodiment intuitions (in Experiments 1–2). These observations (from Western participants) cannot establish universality, but the association of Dualism with ToM suggests its roots are psychological. Thus, the illusory mind–body divide may arise from the very workings of the human mind.

## Introduction

A large literature suggests that people are intuitive Dualists—they tend to consider the mind as ethereal, distinct from the body^1–17^. For example, people believe that one’s psychological traits (e.g., thinking about one’s wife) can persist in the afterlife, without one’s body^18,19^. Conversely, if one’s body were to be duplicated, the replica, people state, would preserve one’s physical characteristics (e.g., a scar), but not one’s knowledge and beliefs^2,20^.

Although most of the experimental evidence for Dualism is obtained from Western participants, non-Western participants likewise contrast minds and bodies^4,5,13,14,17,21–24^. This is in line with the ethnographic record, demonstrating that beliefs in the afterlife (i.e., disembodied self) and in disembodied supernatural beings (e.g., deities, spirits and ghosts) are prevalent across cultures^25,26^. For Western participants, however, intuitive Dualism can be especially detrimental, as it interferes with scientific literacy^10,11,27^, it promotes stigma (e.g., towards psychiatric patients)^8,28,29^ and biases judgements of criminal justice and moral responsibility^30–34^.

Why people might be Dualists, however, is unclear. One possibility is that Dualism is the product of culture—it arises entirely via learning. While learning can, no doubt, propagate Dualism, the learning account fails to explain why intuitive Dualism is so pervasive across cultures. On an alternative account, intuitive Dualism is natural for humans—it arises from two core principles that lie deep within human cognition. One such principle is intuitive physics; the other is theory of mind^35^.

Per intuitive physics, people consider objects as cohesive entities that can only move by immediate contact, and this knowledge manifests in early infancy^36,37^. For example, upon seeing one moving ball contact another (stationary) ball, infants expect the stationary ball to launch immediately. So, if the launch is delayed, infants are surprised—a response evident already in newborns^38^.

Theory of mind (ToM), however, leads us to attribute the actions of agents to their mental states—to their beliefs, knowledge, desires and goals^39–41^. Thus, upon seeing a person reach their hand towards a water bottle, one would spontaneously infer that the person believes there is water in the bottle, and it is this belief that caused their hand to move. This inference, however, blatantly violates intuitive physics, as it presumes that the agent’s hand (a physical object) can move spontaneously, in the absence of contact with another physical objects. And that violation of intuitive physics is bound to elicit cognitive tension. To resolve the dissonance, people might presume that mental states—the causes of the agents’ movement—aren’t physical. Thus, the tension between intuitive ToM and intuitive physics can explain how intuitive Dualism arises naturally.

The hypothesis that Dualism is natural also generates a testable prediction. If Dualism arises (in part) from ToM, then if ToM were to be attenuated, then so should intuitive Dualism^35^.

Recent results from autistic people bear this prediction out^42^. A large literature suggests that autistic people are less adept at reading the minds of others^39,43–53^. If autism attenuates ToM, and ToM begets Dualism, then in people with autism, the mind–body divide ought to be attenuated. Moreover, Dualist intuitions ought to correlate with sensitivity to ToM. This, is exactly what was found^42^. Still, the results from autistic individuals cannot speak to whether this attenuation of the mind–body divide is in fact caused by Dualism.

To further evaluate the origins of Dualism, and its link to ToM, here, we explore systematic gradations in ToM occurring within the neurotypical population—differences between females and males.

Numerous studies have found that males underperform females in ToM tasks. These reports obtain in both adults^54–62^ and children^63–68^ across dozens of languages and societies^69^, including a small-scale one (nomadic pastoralist^63^). The available findings do not establish whether this gender difference is universal or inherent to biological sex^70,71^. Still, one wonders whether this well-established gender difference is linked to Dualism.

If ToM is attenuated in males, and if ToM begets Dualism, then males ought to show weaker Dualism (relative to females) and instead, they ought to lean towards Physicalism (the view of body and mind as one and the same).

Experiments 1–2 explore this prediction. Experiment 1 invites participants to reason about a futuristic scenario whereby it would be possible to create a replica of one’s body; their task is to evaluate which of the donor’s traits will emerge in the replica. Experiment 2 evaluates the converse. Here, participants consider whether some psychological traits persist in the afterlife, after the demise of the body. The critical question is whether intuitions concerning embodiment vary by gender. If males lean towards Physicalism, then they ought to be more likely to consider psychological traits as embodied, hence, as more likely to emerge in the body-replica (and less likely to persist in the afterlife).

**Figure 1:**
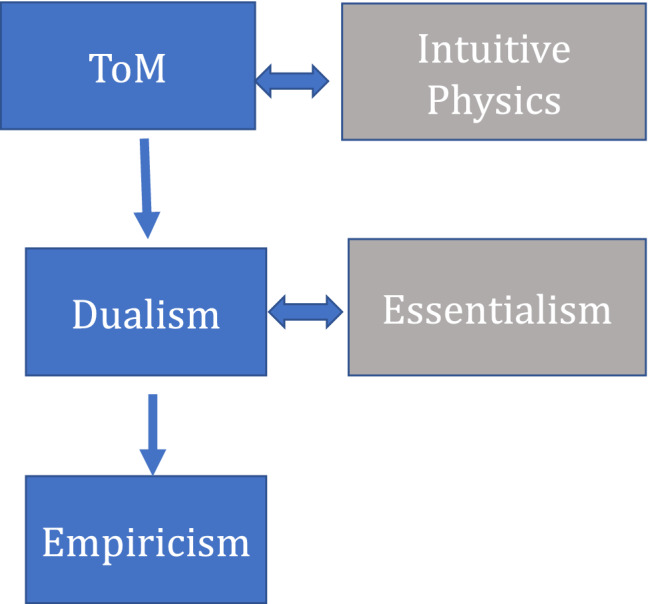
The hypothesized links between ToM, Dualism and Empiricism.

Experiment 3 explores the putative effect of Dualism on another aspect of reasoning—innateness intuitions. Past research suggests that Dualism promotes Empiricism—the belief that psychological traits cannot be innate (due to a Dualism–Essentialism interaction, discussed next)^8,10,72,73^. If males are less Dualist, and Dualism typically begets Empiricism, then males ought to further show weaker Empiricism (see Fig. 1). Experiment 3 examines this prediction. A final analysis evaluates whether the putative gender differences in reasoning about bodies and minds (in Experiments 1–2) are linked to ToM.

Before considering the results, two words of caution are in order. First, the hypothesized difference between males and females with respect to Dualism and Empiricism should only hold inasmuch as males and females differ systematically on their ToM abilities. Our results cannot establish whether differences in ToM arise universally, and if so, why—whether the difference is inherent to biological sex, or whether it arises from culture or other factors (e.g., linguistic ability^74,75^).

Second, all predictions, are relative. Thus, if males are indeed less prone to Dualism and Empiricism, such results do not imply that males are strict Physicalists (i.e., that they consider minds as indistinguishable from bodies) or Nativists. Indeed, males can certainly engage in mind reading—the small gender difference is only found relative to female. Accordingly, our question here, is whether, *compared to females*, males, in this sample, are less inclined towards Dualism and Empiricism, and whether these differences are linked to ToM.

## Results

### Body replication

In Experiment 1, participants (240 Prolific workers) were presented with a list of 80 psychological traits. Half of those traits captured actions and emotions (e.g., walking, anger)—traits that people readily anchor in the body (e.g., legs, face); the other half corresponded to knowledge and beliefs (e.g., i.e., epistemic traits)—traits that people do not readily link to any particular bodily organ (e.g., having a concept of a person; forming sentences). Their task was to indicate whether these traits would emerge in a replica of one’s body.

Since people consider epistemic traits as relatively disembodied, they should view them as less likely to emerge in the replica than emotions and action (hereafter, *non-epistemic* traits), in line with intuitive Dualism. Of interest is whether females should show stronger Dualism than males.

**Figure 2:**
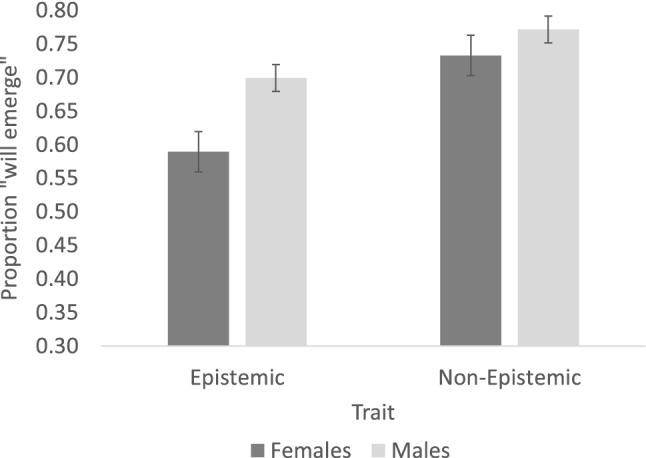
The perceived likelihood that psychological traits will emerge in the body replica. Error bars are standard error of the means (computed over the subjects’ means).

Figure 2 plots the responses. An inspection of the means suggests that, as expected, participants considered epistemic traits as less likely to emerge in the replica relative to non-epistemic traits. Critically, males considered psychological traits as more likely to emerge in the replica than females, and this difference was especially pronounced for epistemic traits.

These observations were evaluated via a generalized linear mixed effects model (response ~ trait * gender + (trait | subject) + (1 | item)), conducted over the binary trial responses (1 = will transfer to the replica; 0 = will not transfer). This model used the binomial response family in R, implemented within the glmer function of the lmer4 package^76^.

The significant effect of Gender (β = 0.76, SE = 0.30, Z = 2.51, p = 0.01) confirmed that males considered psychological traits as more likely to emerge in the replica than females. The significant effect of Trait (β = 0.93, SE = 0.25, Z = 3.68, p < 0.001) also showed that non-epistemic traits were considered more likely to emerge in the replica than epistemic traits. The significant interaction, however, indicated that the effect of Gender was further modulated by Trait type (β = − 0.44, SE = 0.21, Z = − 2.12, p = 0.03).

The (Gender × Trait) interaction was next interrogated by paired comparisons, controlling for the family-wise error rate using the Tukey method (implemented in the emmeans package in R). Results showed that males and females did not differ in their response to non-epistemic traits (ones that people readily anchor in the body: β = − 0.54, SE = 0.31, Z = − 1.72, p > 0.31). But when they considered epistemic traits (ones seen as ethereal), here, males were more likely to state that epistemic traits are likely to emerge in the replica (β = − 0.982, SE = 0.326, Z = − 3.01, p = 0.01). A post-hoc power analysis (conducted using the Wilcoxon-Mann–Whitney two-group test in G*power) indicated that this analysis had sufficient power to capture this contrast of interest (power = 0.889). In addition, the post-hoc tests showed that females considered non-epistemic traits as more likely to emerge than epistemic ones (β = − 1.15, SE = 0.27, Z = − 4.28, p < 0.0001), whereas for males, this difference was just marginally significant (β = − 0.712, SE = 0.28, Z = − 2.55, p = 0.052).

Altogether, these results suggest that males consider epistemic traits as more likely to emerge in a replica of one’s body than females. The selectivity of the gender-difference to epistemic traits suggests that the gender difference likely arises from males’ Physicalist stance, rather than from a simple response bias.

To further test the hypothesis that males are more likely to view the psyche as embodied, Experiment 2 invited the same participants to judge the converse, namely, whether psychological traits are likely to emerge in the afterlife (with task order counterbalanced). If males are less leaning towards Dualism (and more likely to embrace Physicalism), then now, males ought to consider psychological traits as less likely to emerge than females.

### Afterlife

**Figure 3:**
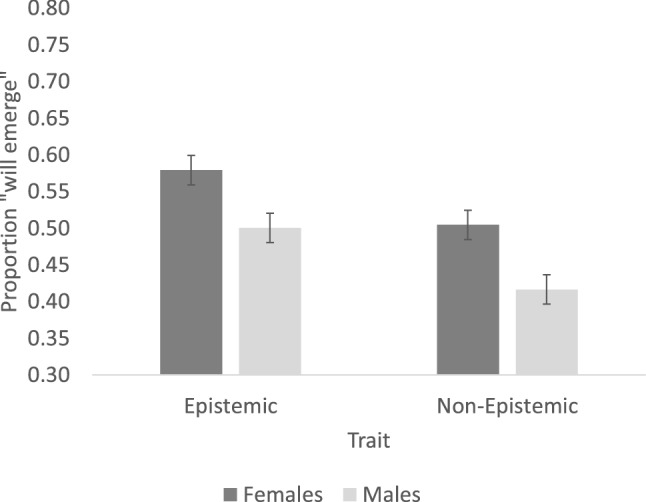
The perceived likelihood that psychological traits will emerge in the afterlife. Error bars are standard error of the means (computed over the subjects’ means).

An inspection of the means (Fig. 3) suggests that, when people reasoned about the afterlife, males considered psychological traits as less likely to emerge (than females). The mixed effect model (described in Experiment 1) yielded a significant main effect of Gender (β = − 0.695, SE = 0.22, Z = − 3.10, p < 0.01), and the power to observe it was 0.850 (α = 0.05). The effect of Trait (β = − 0.49, SE = 0.31, Z = − 1.59, p > 0.11) and the interaction (|Z| < 1) were not significant.

Taken as a whole, Experiments 1–2 suggest that males consider epistemic psychological traits as more likely to emerge in a manipulation that preserves one’s body (i.e., body-replication), but they view (all) psychological traits as less likely to emerge upon the body’s demise, in the afterlife. The dissociation between the two tasks—depending on whether the task gauges the emergence of the psyche in the body (in the replica), or in its absence (in the afterlife)—makes it clear that the shift in response specifically concerns the perception of the psyche as embodied. The gender difference thus demonstrates that males consider the psyche as more strongly embodied than females.

To further explore the link between gender and the mind–body divide, in Experiment 3, we examine yet another consequence of Dualism, namely, innateness intuitions. If ToM begets Dualism, then males and females ought to also differ on that dimension.

### Innateness intuitions

Past research has shown that people (mostly, WEIRD^77^ participants) are intuitive Empiricists—they believe that knowledge and beliefs are unlikely to be innate^78,79^. This aversion to innateness is selective—people do not simply reject all aspects of innateness. In fact, they maintain that emotions, actions, and sensations are innate^73,78^. It is specifically epistemic states—knowledge and beliefs—that they consider as arising strictly from experience^73,78^.

Taken at face value, this Empiricist stance could arise from multiple sources, including instinct blindness^80^, mind opacity^81^, and concerns with social harm^82^. But there is evidence that Dualism contributes to our Empiricist intuitions^27,83^.

Past research has shown that innateness intuitions are guided by Essentialism—the belief that living things are what they are because they possess some innate, immutable essence that resides in their bodies^84–86^. Per Dualism, however, epistemic states (e.g., knowledge, beliefs) are ethereal, distinct from the body. It thus follows that epistemic states cannot be innate^27,83^.

Past research has shown that innateness intuitions are indeed linked to embodiment intuitions. When presented with evidence that a psychological trait is embodied, participants are more likely to consider that trait as innate (compared to when told that the same trait has no known anchoring in the body)^8,10,72^.

If Dualism indeed begets Empiricism, then a reduction in Dualism ought to attenuate one’s Empiricist stance. This prediction is borne out by two pieces of evidence. First, when neurotypical participants are primed to consider bodies and minds alike (i.e., towards Physicalism), their empiricist stance declines (temporarily)^10,72^. The second piece of evidence comes from people with autism—a condition that is known to attenuate ToM^39,43–53^. If ToM promotes Empiricism (due to its role in Dualism), then people with ASD should be less likely to lean towards Empiricism, and instead favor Nativism (relative to neurotypical people). This is precisely what was found^42^.

Following this logic, one would expect gender to elicit similar effects (see Fig. 1). In particular, if (a) males show weaker ToM, (b) ToM begets Dualism, and (c) Dualism encourages Empiricism, then, not only should males (i) exhibit weaker Dualism (as shown in Experiments 1–2) but also (ii) weaker Empiricism.

To test this last prediction, Experiment 3 invited a new sample of males and females (N = 242) to reason about the innateness of psychological traits (the same traits as in Experiments 1–2). We expect males to consider psychological traits as more likely to be innate than females.

**Figure 4:**
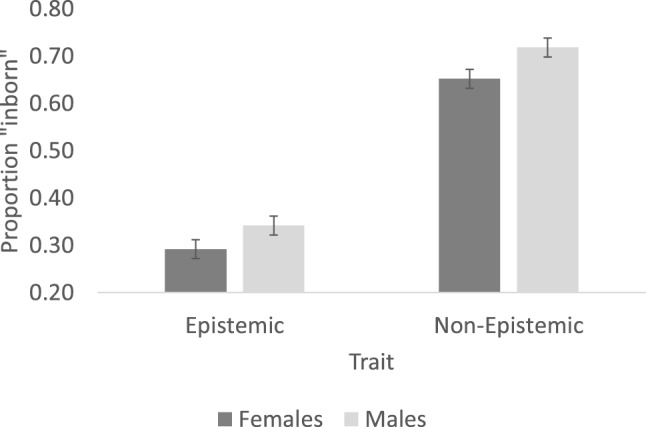
The perceived likelihood that psychological traits are inborn. Error bars are standard error of the means (computed over the subjects’ means).

Figure 4 plots participants’ innateness intuitions. An inspection of the means suggests that, compared to females, males were more likely to consider psychological traits as innate. Additionally, epistemic traits were considered as less likely to be innate than non-epistemic traits.

In line with these observations, a mix-effect model (described in Experiment 1) yielded a significant main effect of Gender (β = 0.424, SE = 0.16, Z = 2.59, p < 0.01) and of Trait (β = 2.54, SE = 0.33, Z = 7.59, p < 0.001); the interaction (Trait × Gender) was not significant (β = 0.218, SE = 0.15, Z = 1.41, p > 0.16). The power to detect the main effect of Gender was 0.88.

To further evaluate whether people did, in fact, reject the innateness of Epistemic traits, the mean response was next compared against chance using a mixed effect model (0, in log odds; response ~ (1 | subject) + (1 | item)). Results confirmed that non-epistemic traits were rated significantly above chance (β = 1.32, SE = 0.286, Z = 4.61, p < 0.001), suggesting that people did consider these traits as innate. But when people evaluated epistemic traits, here, responses were significantly lower than chance (β = − 1.19, SE = 0.20, Z = − 5.95, p < 0.001), suggesting that people denied that epistemic traits are innate.

These results confirm that participants are indeed Empiricist—they selectively deny that knowledge and beliefs are possibly innate^8,10,72,73^. Males, however, are less likely to exhibit this bias than females.

### Is dualism linked to ToM?

Experiments 1–3 demonstrate that males are less Dualist than females, and perhaps for this reason, they are also more likely to view psychological traits as innate. These systematic gender differences are precisely what would be predicted by the hypothesis that ToM begets Dualism, and that males’ ToM is slightly weaker than females’, in line with past research^54–68^. Still, the results discussed thus far do not address the source of the gender difference: is it linked to ToM?

To address this question, we first compared the males and females in this sample on a ToM test; we next evaluated whether ToM is linked to reasoning about bodies and minds.

In the ToM task (adapted from^87^), participants were presented with brief vignettes featuring a protagonist who either does or doesn’t hold a certain belief. In half of the trials, the belief was false, but the protagonist was likely to hold it (e.g., “Lisa believes Jacob is sleeping”; Lisa is likely to hold this belief as she last saw Jacob asleep on the beach, but alas, this belief is false—in reality, Jacob has since gone swimming). The other half featured true beliefs that the protagonist was unlikely to hold (e.g., “the girls believe the ice-cream in the fridge is melted”; this belief is actually true, as a power outage occurred overnight, but the girls have no reason to hold that belief, as they were meanwhile sleeping). Participants were asked to quickly evaluate whether or not the protagonist holds this belief. Sensitivity to ToM was gauged by response accuracy speed. This analysis was applied to the entire sample of Experiments 1–2 (excluding one non-binary participant and one inattentive participant whose mean response accuracy on the ToM test was 0.05; N = 238).

**Figure 5:**
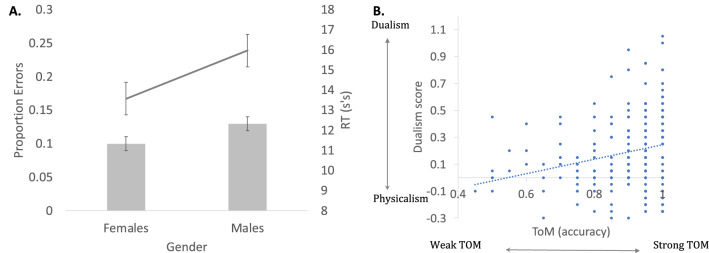
ToM response (the bar graph captures errors; response times are indicated by the line graph) (**A**); and the correlation between ToM accuracy and Dualism scores (**B**).

Results (Fig. 5A) showed that, compared to females, males in this sample indeed had greater difficulty reasoning about the minds of others, as their accuracy was significantly lower (β = − 0.33, SE = 0.16, Z = − 2.03, p = 0.04), and their response time was higher (t(236) = 1.97, p = 0.05)

To determine whether ToM performance is linked to reasoning about minds and bodies, we next assessed participants’ Dualist stance by computing their “Dualism score”—their tendency to consider epistemic traits as more likely to emerge in the afterlife, and less likely to emerge in the replication task (∆(epistemic-nonepistemic)_Afterlife_ + ∆(nonepistemic-epistemic)_Replication_). This analysis excluded the outliers on the Dualism tasks (as described in Experiments 1–2).

Dualism scores were next correlated with ToM performance in Experiments in 1–2 (where Dualism was assessed). The correlation between Dualism and ToM accuracy was significant (r(215) = 0.24, p < 0.001 see Fig. 5b): the better were participants able to reason about the minds of others (i.e., higher ToM accuracy) the more Dualist they were (i.e., larger Dualism scores). These results suggest that reasoning about bodies-and minds is linked to the ability to reason about the minds of others.

## Discussion

People tend to consider the mind as ethereal, distinct from the body. This intuitive Dualist stance has been demonstrated in adults and children, in Western and non-Western participants^1–18,20–24,88,89^, and its consequences on reasoning are widespread^8,10,11,27–34^.

Why people are putative Dualists, however, is unclear. In particular, one wonders whether Dualism arises only by cultural transmission, or whether the illusion of the mind–body divide can also emerge naturally, from ToM^35^.

To address this question, here, we investigated whether individual differences in ToM capacities, occurring within the neurotypical population—between males and females^54–69^—are linked to Dualism. Experiments 1–2 show that this is indeed the case.

Males, in this sample, considered the psyche as more strongly embodied than females: they believed that epistemic states are more likely to emerge in a replica of one’s body (in Experiment 1) and that psychological traits are less likely to persist upon the body’s demise, in the afterlife (in Experiment 2). Experiment 3 further showed that males are also more likely to consider psychological traits as innate—this is expected by past findings, suggesting that Dualism begets Empiricism^8,10,72,73^.

A follow-up analysis has confirmed that these differences in reasoning about bodies and minds are linked to ToM. Not only did males in this sample score lower than females on ToM, but their ToM scores correlated with their Dualist intuitions.

As noted, these results ought to be interpreted with caution, as the gender differences observed here may not hold universally, and it certainly does not speak to the reasoning of any individual person. And indeed, ToM abilities demonstrably depend on multiple factors, including linguistic experience^74,75^ and culture^90,91^. But inasmuch as females show superior ToM, they ought to lean towards Dualism and Empiricism. Dualism, then, is linked to ToM.

Whether ToM in fact promotes Dualism is more difficult to determine. Still, the present findings (from neurotypical males and females) complement past research from autistic individuals^42^. In both cases, lower ToM performance (in males, and in people with ASD) is linked to weaker Dualism and stronger Nativism (relative to females and the NT population, respectively). This association (between ToM and Dualism) falls short of establishing causation. Nonetheless, the convergence between the two sets of findings—from neurotypical and autistic people—is striking. This outcome is in line with the hypothesis that ToM begets Dualism. The illusion of the mind–body divide, then, may not be solely a cultural construct. Rather, Dualism might be grounded in a core psychological mechanism that lies deep within the human mind.

Dualism is further significant because it demonstrably stands in the way of scientific literacy and the understanding of public health. Specifically, Dualism promotes false beliefs about neuroscience and innateness^10,11,27^, it engenders misconceptions about psychiatric disorders^8,28,29^, and meddles with reasoning about moral responsibility and criminal justice^30–34^. Understanding the origins of Dualism may help combat its many tolls on reasoning and shed light on human nature.

## Methods

### Experiments 1–2 (replication/afterlife)

#### Participants

Participants were 240 Prolific workers. They were native English speakers, neurotypical, with no known diagnosis of language- reading disorders or ASD. Each experiment recruited an equal number of males and females; one participant self-identified as non-binary was excluded from the analysis (for participants’ demographics and country of origin, see Figs. S1-2). Sample size was informed by pilot work, suggesting that the selected sample size is sufficient to attain a power of 0.8 at the 0.05 alpha level.

#### Data exclusion

The analyses of Experiments 1–2 excluded participants whose mean response fell 2 SD beyond the cell mean. This procedure removed 15 participants from Experiment 1 (6 females; one additional participant, identified as nonbinary, was likewise excluded); and 9 participants (all females) from Experiment 2. An analysis of the entire sample yielded similar conclusions.

#### Materials and tasks

Participants took part in four tasks. First, participants were given the two “Dualism” tasks—body replication and afterlife (counterbalanced for order). Next, they were administered the theory of mind task. Finally, participants were given the autism Quotient task.

The Dualism tasks featured a list of 80 psychological traits (from^10,78^). Half of those traits captured knowledge and beliefs—hereafter, “epistemic traits” (e.g., having a concept of a person); the other half correspond to actions and emotions (e.g., squatting, fear), hereafter, *non-epistemic* traits. These traits were arranged in two counterbalanced lists. Each participant performed each task with a different item list, such that, across participants, all traits were presented equally in both tasks, and within each task, each trait only appeared once.

In Experiment 1, participants were invited to suppose it was possible to grow a replica of the body of an adult human donor. The replica preserves every aspect of the human body and brain. With this in mind, participants were asked to determine whether a given trait would likely emerge in the replica. In Experiment 2, participants were invited to judge whether each trait is likely to persist in the afterlife. For the full list of materials and instructions in Experiments 1–2 see SM, Appendices I–II.

After the two Dualism probes, participants were given the ToM task. In the ToM test (adapted from^87^), participants were randomly presented with 20 short vignettes. Each vignette features a protagonist who either does or does not hold a certain belief. The belief, in turn, was either true or false. In so doing, we sought to dissociate the presence of true/false belief with its veracity. Thus, in half of the vignettes, the belief was false, and the protagonist was likely to hold that belief (under the circumstances detailed in the vignette), whereas in the others, the belief was true, but the protagonist was unlikely to hold it. Participants were instructed to quickly determine whether the probe sentence was true or false (for the full materials and instructions, see SM, Appendix III).

Finally, participants were given the AQ (from^55^; this test asks participants to respond to fifty short sentences, related to their social skills, attention switching, attention to detail, communication, and imagination.

### Experiment 3 (Innateness intuitions)

#### Participants

Experiment 3 was administered to 242 participants. Of those, 120 were Prolific workers (60 males); the remaining 122 participants with students and the department of Psychology at Northeastern University who took part in the experiment for course credit. Because the population of Psychology students at Northeastern is heavily female-dominated, most Northeastern participants were females (N = 94). Sample size was set to match Experiments 1–2 (for participants’ demographics and country of origin, see Figs. S1–2).

#### Data exclusion

As in previous experiments, the analysis of Experiment 3 excluded participants whose mean response fell 2 SD beyond the mean. 21 participants (16 females) were excluded from Experiment 3. An analysis of the entire sample yielded similar conclusions.

#### Materials

All participants took part in three tasks. First, participants performed the “innateness” task, next they performed the ToM task, and finally the AQ evaluation. The subset of Psychology students also took part in the two Dualism tasks (as in Experiments 1–2), administered prior to the Innateness task.

The innateness task featured the same list of 80 psychological traits from Experiments 1–2 (with order randomized). These items were designed to capture characteristics that are likely to hold across cultures; epistemic traits, specifically, were selected from a list of traits that have been widely documented in small scale societies (adapted from the Appendix in^82^).

Participants evaluated whether these traits are inborn in humans. Participants were told that “inborn traits are ones that develop in humans spontaneously. Some of these traits (e.g., having five fingers) are present at birth, but others (e.g., facial hair in men) can appear later in development. All inborn traits, however, emerge in the typical course of development, even if an individual has never had the opportunity to witness these behaviors in other people”. Participants were asked to give a binary response as to whether each trait is inborn in humans. The full lists of materials are presented in the SM, Appendix I–II.

All materials and procedures were reviewed and approved by the Institutional Review Board at Northeastern University; all methods were performed in accordance with those guidelines and regulations. An informed consent form was obtained from all participants or their parents/legal guardians.

## Supplementary Information

Supplementary Information.

Supplementary Table 1.

## Data Availability

All data are made available in the SM file, attached.
