# Supplementary material for: The illusion of the mind–body divide is attenuated in males
Source: Sci Rep. 2023 Apr 24;13:6653. doi: 10.1038/s41598-023-33079-1 (PMC10126148; doi:10.1038/s41598-023-33079-1)
Supplement: Supplementary file 1 — Supplementary Information. [file 41598_2023_33079_MOESM1_ESM.docx]

**_­­­_The illusion of the mind-body divide is attenuated in males**

**SM**

Iris Berent

Northeastern University

**Address for correspondence:**

Dr. Iris Berent

Department of Psychology

Northeastern University

125 Nightingale Hall

360 Huntington Ave.

Boston MA 02115 USA

i.berent@northeastern.edu

Table S1. The characteristics of participants in Experiments 1-3.

| ***Experiment*** | ***Gender*** | ***N*** | ***Age*** | | ***Education level*** | | | | | |
| --- | --- | --- | --- | --- | --- | --- | --- | --- | --- | --- |
|  |  |  | *Mean* | *SD* | *Highschool/GED* | *Associate* | *Bachelors* | *MA* | *Doctorate* | *Other* |
| 1-2 | Female | 119 | 37.19 | 13.53 | 36 | 4 | 40 | 13 | 3 | 3 |
|  | Male | 120 | 40.96 | 33.55 | 31 | 9 | 45 | 10 | 1 | 4 |
| 3 | Female | 154 | 26.19 | 12.15 | 106 | 5 | 32 | 9 | 1 | 1 |
|  | Male | 88 | 31.51 | 13.76 | 51 | 4 | 23 | 7 | 1 | 2 |

Note: Education level lists the highest level of education attained; the figures reflect the percentage of participants who selected each category.

Table S2. The county origins of participants in Experiments 1-3

| ***Experiment*** | ***Gender*** | ***Country*** | | | | | | |
| --- | --- | --- | --- | --- | --- | --- | --- | --- |
|  |  | United Kingdom | United States | Canada | South Africa | Ireland | | Other |
| 1-2 | *Female* | 67 | 1 | 12 | 13 | 3 | | 3 |
|  | *Male* | 53 | 2 | 13 | 21 | 8 | | 3 |
|  | *Total* | 60 | 1 | 12 | 17 | 6 | | 3 |
| 3 | *Female* | 19 | 61 | 6 | 10 | 1 | | 1 |
|  | *Male* | 31 | 35 | 15 | 9 | 2 | | 7 |
|  | *Total* | 24 | 52 | 9 | 10 | 1 | | 3 |
|  |  |  |  |  |  |  | |  |
|  |  | | | | | | | |
| *Note:* Country of origin is inferred from participants’ IP address; all figures capture percentages; "other" includes Mexico, Poland, Hungary, France, Chile, New-Zealand, Korea, Spain (each country accounts for less than 2% of the participants) | | | | | | |  |  |

**Appendix 1**

**The list of psychological traits in Experiments 1-3**

| **Number** | **Category** | | **Trait** |
| --- | --- | --- | --- |
| 1. | Non-epistemic | emotion | Anger in response to hostility |
| 2. | Non-epistemic | emotion | Love for one’s family |
| 3. | Non-epistemic | emotion | Contentment with one’s life |
| 4. | Non-epistemic | emotion | Disgust by feces |
| 5. | Non-epistemic | emotion | Excitement towards an opportunity |
| 6. | Non-epistemic | emotion | Fear of danger |
| 7. | Non-epistemic | emotion | Happiness at the birth of one’s child |
| 8. | Non-epistemic | emotion | Joy of being |
| 9. | Non-epistemic | emotion | Pride in one’s accomplishments |
| 10. | Non-epistemic | emotion | Sadness from a friend’s death |
| 11. | Non-epistemic | emotion | Shame from one’s shortcomings |
| 12. | Non-epistemic | emotion | Surprise at an unexpected event |
| 13. | Non-epistemic | emotion | Trust in one’s family |
| 14. | Non-epistemic | emotion | Jealousy towards a lover |
| 15. | Non-epistemic | emotion | Envy at a competitor’s success |
| 16. | Non-epistemic | emotion | Empathy towards a person in need |
| 17. | Non-epistemic | emotion | Admiration for wisdom |
| 18. | Non-epistemic | emotion | Pain from witnessing illness and death |
| 19. | Non-epistemic | emotion | Hope for a better future |
| 20. | Non-epistemic | emotion | Affection towards others |
| 21. | Non-epistemic | motor | Gripping objects by hand |
| 22. | Non-epistemic | motor | Sitting down to relax |
| 23. | Non-epistemic | motor | Walking to move around |
| 24. | Non-epistemic | motor | Running when in a hurry |
| 25. | Non-epistemic | motor | Kicking with one’s feet |
| 26. | Non-epistemic | motor | Lifting objects with hands |
| 27. | Non-epistemic | motor | Stretching one’s muscles |
| 28. | Non-epistemic | motor | Licking with one’s tongue |
| 29. | Non-epistemic | motor | Yawning when tired |
| 30. | Non-epistemic | motor | Breathing heavily after exertion |
| 31. | Non-epistemic | motor | Squatting down |
| 32. | Non-epistemic | motor | Trembling at cold temperatures |
| 33. | Non-epistemic | motor | Tickling a child to make them laugh |
| 34. | Non-epistemic | motor | Touching other people with one’s hands |
| 35. | Non-epistemic | motor | Smelling the scent of food |
| 36. | Non-epistemic | motor | Sleeping to restore one’s energy |
| 37. | Non-epistemic | motor | Seeing objects with one’s eyes |
| 38. | Non-epistemic | motor | Sniffling when one has a cold |
| 39. | Non-epistemic | motor | Crying at sad events |
| 40. | Non-epistemic | motor | Dancing at to a rhythm |
| 41. | Epistemic | | Recalling past events |
| 42. | Epistemic | | Judging one’s options |
| 43. | Epistemic | | Distinguishing between right and wrong |
| 44. | Epistemic | | Reflecting on one’s past and future |
| 45. | Epistemic | | Having self control of one’s own behavior |
| 46. | Epistemic | | Speculating about the possible outcomes of events |
| 47. | Epistemic | | Making jokes |
| 48. | Epistemic | | Thinking about magic |
| 49. | Epistemic | | Using metaphors |
| 50. | Epistemic | | Mourning the dead |
| 51. | Epistemic | | Observing rituals |
| 52. | Epistemic | | Overcoming a fear |
| 53. | Epistemic | | Recognizing taboos |
| 54. | Epistemic | | Recognizing relations among kin |
| 55. | Epistemic | | Interpreting others' behaviors |
| 56. | Epistemic | | Symbolic reasoning |
| 57. | Epistemic | | Making comparisons |
| 58. | Epistemic | | Keeping track of time |
| 59. | Epistemic | | Planning for the future |
| 60. | Epistemic | | Recognizing melodies |
| 61. | Epistemic | | Keeping track of people’s age |
| 62. | Epistemic | | Forming sentences |
| 63. | Epistemic | | Forming words |
| 64. | Epistemic | | Abstract reasoning |
| 65. | Epistemic | | Having preferences concerning aesthetics |
| 66. | Epistemic | | Attributing human qualities to inanimate objects |
| 67. | Epistemic | | Having a belief in the super-natural |
| 68. | Epistemic | | Having beliefs about fortune and misfortune |
| 69. | Epistemic | | Devising classification of body parts |
| 70. | Epistemic | | Having classification of animals |
| 71. | Epistemic | | Having classification of plants |
| 72. | Epistemic | | Having classification of weather |
| 73. | Epistemic | | Devising methods to heal the sick |
| 74. | Epistemic | | Having a contrast between 'general' and 'particular' |
| 75. | Epistemic | | Having a logical notion of 'and' |
| 76. | Epistemic | | Having a logical notion of 'not' |
| 77. | Epistemic | | Forming myths |
| 78. | Epistemic | | Having a concept of 'person' |
| 79. | Epistemic | | Having a preference for one's own children and close kin |
| 80. | Epistemic | | Having norms about trade |

**Appendix II: Instructions in Experiments 1-3**

**Instructions in the body replication task (Experiment 1)**

In what follows, we are asking you to reason about a hypothetical scenario.

Suppose it were possible to grow a replica of the body of an adult human donor. The replica preserves every aspect of the human body and brain. In particular, suppose that the body of the replica looks and works precisely like a normal human body. Similarly, the brain replica is identical in all respects to the brain of the adult donor.

Below is a list of various traits that define the human donor. Will these traits emerge in its replica?

Please indicate your answer as either 1=yes, this trait will emerge in the replica OR 2=no, this trait will not emerge in the replica. Thank you!


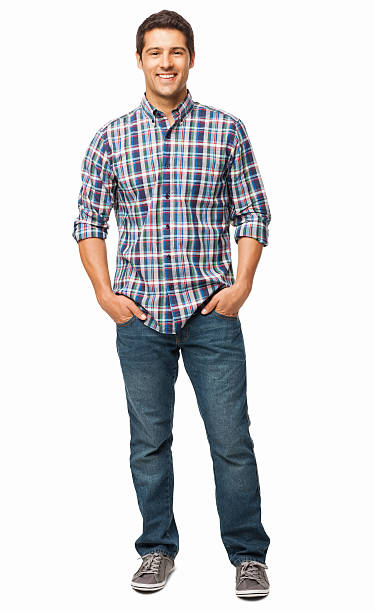


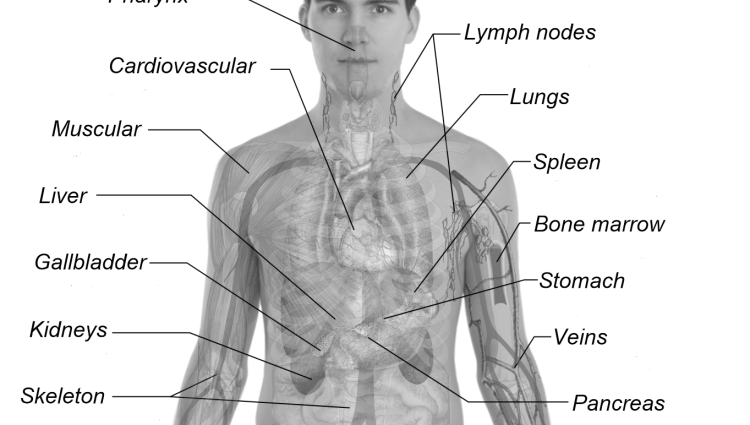


**Instructions in the afterlife task (Experiment 2)**

In this experiment, we examine people’s beliefs about the afterlife. Whether or not there is an afterlife, is of course unknown. But for the purpose of this experiment, we invite you to assume that after people die, they do continue to exist in some capacity. Your task is to reason about which human traits are likely to be maintained in the afterlife.

Below is a list of human traits. Then, please determine the following: if an afterlife exists, will these traits emerge in the afterlife?

Please indicate your answer as either 1=yes, this trait will emerge in the afterlife OR 2=no, this trait will not emerge in the afterlife.  Thank you!

**Instructions in the innateness task (Experiment 3)**

In this experiment, you will read a list of human traits. Please read each description carefully. Then, please determine whether or not these traits are **inborn** in humans.

Inborn traits are ones that develop in humans spontaneously. Some of these traits (e.g., having five fingers) are present in birth, but others (e.g., facial hair in men) can appear later in development.  All inborn traits, however, emerge in the typical course of development, even if an individual has never had the opportunity to witness these behaviors in other people.  Are each of the traits below inborn in humans?

Please indicate your answer as either 1=yes, this trait is inborn in humans OR 2=no, this trait is not inborn in humans.

Thank you!

**Instructions and materials in ToM task (Experiments 1-3)**

**Instructions**

We typically go about our everyday life guided by our beliefs about how things are, and about what other people think. Sometimes these beliefs are true; but at times, they are not.

In this experiment, you will be presented with short stories, describing a person and their beliefs. We ask you to indicate whether this belief is true or false. Please respond as quickly and accurately as you can.

 Thank you!

**Appendix III**

**Materials and instructions for the ToM task (in Experiments 1-3)**

| # | Story | Answer |
| --- | --- | --- |
|  | The morning of high school dance Sarah placed her high heel shoes under her dress and then went shopping. That afternoon, her sister borrowed the shoes and later put them under Sarah's bed. *Sarah gets ready assuming her shoes are under the dress.* | yes |
|  | John told Mary that he had lost his keys, and the two of them go searching. While John looks outside, Mary finds the keys, but since she’s in a hurry, she just places them on the table at the entrance and leaves. *When John returns, he expects to see the keys on the table.* | no |
|  | Expecting the game to be suspended because of the rain, the Garcia family took the subway home. The score was tied, 3-3, and on their way home, the family had no internet connection. During their commute the rain stopped and the game soon ended with a score of 5-3. *The Garcia family arrives home believing the score is 5-3.* | no |
|  | Susie parked her sports car in the driveway. In the middle of the night, Nathan moved her car into the garage to make room for his minivan. When Susie wakes up, *she expects to see her car in the driveway.* | Yes |
|  | When Lisa left Jacob, he was deep asleep on the beach. A few minutes later a wave woke him. Seeing Lisa was gone, Jacob decided to go swimming. *Lisa now believes that Jacob is sleeping.* | Yes |
|  | Larry chose a debated topic for his class paper due on Friday. The news on Thursday indicated that the debate had been solved, but Larry never read it. *When Larry writes his paper he thinks the debate has been solved.* | No |
|  | A window wiper was commissioned by a CEO to wipe an entire building. He finished the right side, but his platform gets stuck before he could do the left side, so he reports the problem to the CEO. Overnight, the cleaner comes up with a solution, and early in the morning, he diligently returns to finish the job. *When the CEO comes to work at noon, he expects to see all of the windows are cleaned.* | No |
|  | Sally and Greg called ahead of time to make a reservation for the back-country cabin. The park ranger forgot to write down the reservation and two other hikers got to the cabin first. *When the hikers arrive, they expect the other hikers to be in their room.* | No |
|  | Rather than driving to work, today Amy decided to walk. When George woke up, he saw her car in the drive. Amy’s room was quiet and dark. George knows that when Amy is sick, she lies down in a dark room. *George expects Amy is in her room, sick with migraine.* | yes |
|  | Laura didn't have time to braid her horse’s mane before going to camp. While she was at camp, William brushed Laura's horse and braided the horse’s mane for her. *Laura returns assuming that her horse's hair isn't braided.* | Yes |
|  | At night a bear broke into a cooler near a tent and drank the soda. Five hours later, the campers woke up and went to their cooler for breakfast. *In the cooler, the campers expect to find soda.* | Yes |
|  | Anne made lasagna in the blue dish. After Anne left, Ian came home and ate the lasagna. Then he filled the blue dish with spaghetti and replaced it in the fridge. *Anne thinks the blue dish contains spaghetti.* | no |
|  | Jenny put her chocolate away in the cupboard. Then she went outside. Alan moved the chocolate from the cupboard into the fridge. Half an hour later, Jenny came back inside. *Jenny expects to find her chocolate in the cupboard.* | yes |
|  | The weather was so warm today that all the tulips in Pam's backyard suddenly bloomed. The tulips next to Pam's office still have not yet flowered, though. Pam has been at work all day. *Driving home after work, Pam supposes her tulips have not bloomed.* | Yes |
|  | When the class' science test was handed back, Shannon was mistakenly given Adam's test. A large B was written on the front of Adam's test, but Shannon's actual grade was an A. *Shannon believes she received a B on the exam.* | yes |
|  | Every day Jill goes to the coffee shop on the corner and orders a latte, her favorite drink. Today, the cashier misunderstands Jill and prepares a mocha instead. *Jill thinks her drink will taste like a mocha.* | No |
|  | Hopeful to catch a prize fish, George went fishing. That afternoon, he saw his fishing line bend over as if he had caught a big fish. Actually, George’s fishing pole had snagged a small tire. *At the end of the fishing line, George expects to see a tire.* | No |
|  | The girls left ice cream in the freezer before they went to sleep. Overnight, the power to the kitchen was cut and the ice cream melted. *When they get up the girls believe the ice cream is melted.* | No |
|  | Ken told Andrea that he was going shopping for sandals. At the shoe store, Ken noticed a very nice pair of boots on sale, and bought them instead. *When he meets Andrea, she believes Ken's shoe store bag contains boots*. | No |
|  | Jeff is colorblind, so he cannot tell subtle differences in color. To help him dress up, his housekeeper normally keeps the pink and white shirts in different drawers, but this week, she is sick, and her replacement placed the white shirts at the bottom drawer, where the pink shirts usually are. *When Jeff reaches out to the bottom drawer and puts on a shirt, he believes the shirt is pink.* | yes |

**Instructions and materials in the AQ task** (from [56] )

For each statement below, choose one response that best describes how strongly that statement applies to you:

responses are given as: Definitely Agree; Slightly Agree; Slightly Disagree; Definitely Disagree

1. I prefer to do things with others rather than on my own.

2. I prefer to do things the same way over and over again.

3. If I try to imagine something, I find it very easy to create a picture in my mind.

4. I frequently get so strongly absorbed in one thing that I lose sight of other things.

5. I often notice small sounds when others do not.

6. I usually notice car number plates or similar strings of information.

7. Other people frequently tell me that what I’ve said is impolite, even though I think it is polite.

8. When I’m reading a story, I can easily imagine what the characters might look like.

9. I am fascinated by dates.

10. In a social group, I can easily keep track of several different people’s conversations.

11. I find social situations easy.

12. I tend to notice details that others do not.

13. I would rather go to a library than to a party.

14. I find making up stories easy.

15. I find myself drawn more strongly to people than to things.

16. I tend to have very strong interests, which I get upset about if I can’t pursue.

17. I enjoy social chitchat.

18. When I talk, it isn’t always easy for others to get a word in edgewise.

19. I am fascinated by numbers.

20. When I’m reading a story, I find it difficult to work out the characters’ intentions.

21. I don’t particularly enjoy reading fiction.

22. I find it hard to make new friends.

23. I notice patterns in things all the time.

24. I would rather go to the theater than to a museum.

25. It does not upset me if my daily routine is disturbed.

26. I frequently find that I don’t know how to keep a conversation going.

27. I find it easy to “read between the lines” when someone is talking to me.

28. I usually concentrate more on the whole picture, rather than on the small details.

29. I am not very good at remembering phone numbers.

30. I don’t usually notice small changes in a situation or a person’s appearance.

31. I know how to tell if someone listening to me is getting bored.

32. I find it easy to do more than one thing at once.

33. When I talk on the phone, I’m not sure when it’s my turn to speak.

34. I enjoy doing things spontaneously.

35. I am often the last to understand the point of a joke.

36. I find it easy to work out what someone is thinking or feeling just by looking at their face.

37. If there is an interruption, I can switch back to what I was doing very quickly.

38. I am good at social chitchat.

39. People often tell me that I keep going on and on about the same thing.

40. When I was young, I used to enjoy playing games involving pretending with other children.

41. I like to collect information about categories of things (e.g., types of cars, birds, trains, plants).

42. I find it difficult to imagine what it would be like to be someone else.

43. I like to carefully plan any activities I participate in.

44. I enjoy social occasions.

45. I find it difficult to work out people’s intentions.

46. New situations make me anxious.

47. I enjoy meeting new people.

48. I am a good diplomat.

49. I am not very good at remembering people’s date of birth.

50. I find it very easy to play games with children that involve pretending.
